# The impact of response time reliability on CPR incidence and resuscitation success: a benchmark study from the German Resuscitation Registry

**DOI:** 10.1186/cc10566

**Published:** 2011-11-24

**Authors:** Jürgen Neukamm, Jan-Thorsten Gräsner, Jens-Christian Schewe, Martin Breil, Jan Bahr, Ulrich Heister, Jan Wnent, Andreas Bohn, Gilbert Heller, Bernd Strickmann, Hans Fischer, Clemens Kill, Martin Messelken, Berthold Bein, Roman Lukas, Patrick Meybohm, Jens Scholz, Matthias Fischer

**Affiliations:** 1 Department of Anesthesiology and Intensive Care, Klinik am Eichert, Eichertstraße 3, D-73035 Göppingen, Germany; 2 Department of Anesthesiology and Intensive Care Medicine, University Hospital Schleswig-Holstein, Campus Kiel, Schwanenweg 21, D-24105 Kiel, Germany; 3 Department of Anesthesiology and Intensive Care Medicine, University Hospital Bonn, Sigmund-Freud-Street 25, D-53105 Bonn, Germany; 4 Department of Anesthesiology, Emergency Medicine and Intensive Care University Hospital Göttingen, Robert-Koch-Strasse 40, D-37099 Göttingen, Germany; 5 Department of Anesthesiology and Intensive Care Medicine, University Hospital Schleswig-Holstein, Campus Lübeck, Ratzeburger Allee 160, D-23538 Lübeck, Germany; 6 Department of Anesthesiology and Intensive Care Medicine, University Hospital Münster, D-48149 Münster, Germany; 7 Department of Anesthesiology and Intensive Care, Klinikum Ravensberg, Halle (Westfalen), Emergency Medical Services System of the County of Gütersloh, Winnebrockstraße 1, D-33790 Gütersloh, Germany; 8 Department of Anesthesiology and Intensive Care Medicine, University Hospital Tübingen, Hoppe-Seyler-Straße 3, D-72076 Tübingen, Germany; 9 Department of Anesthesiology and Intensive Care Medicine, University Hospital Marburg, Baldingerstrasse, D-35043 Marburg, Germany

## Abstract

**Introduction:**

Sudden cardiac arrest is one of the most frequent causes of death in the world. In highly qualified emergency medical service (EMS) systems, including well-trained emergency physicians, spontaneous circulation may be restored in up to 53% of patients at least until admission to hospital. Compared with these highly qualified EMS systems, markedly lower success rates are observed in other systems. These data clearly show that there are considerable differences between EMS systems concerning treatment success following cardiac arrest and resuscitation, although in all systems international guidelines for resuscitation are used. In this study, we investigated the impact of response time reliability (RTR) on cardiopulmonary resuscitation (CPR) incidence and resuscitation success by using the return of spontaneous circulation (ROSC) after cardiac arrest (RACA) scores and data from seven German EMS systems participating in the German Resuscitation Registry.

**Methods:**

Anonymised patient data after out-of-hospital cardiac arrest gathered from seven EMS systems in Germany from 2006 to 2009 were analysed with regard to socioeconomic factors (population, area and EMS unit-hours), process quality (RTR, CPR incidence, special CPR measures and prehospital cooling), patient factors (age, gender, cause of cardiac arrest and bystander CPR). End points were defined as ROSC, admission to hospital, 24-hour survival and hospital discharge rate. χ^2 ^tests, odds ratios and the Bonferroni correction were used for statistical analyses.

**Results:**

Our present study comprised 2,330 prehospital CPR patients at seven centres. The incidence of sudden cardiac arrest ranged from 36.0 to 65.1/100,000 inhabitants/year. We identified two EMS systems (RTR < 70%) that reached patients within 8 minutes of the call to the dispatch centre 62.0% and 65.6% of the time, respectively. The other five EMS systems (RTR > 70%) reached patients within 8 minutes of the call to the dispatch centre 70.4% up to 95.5% of the time. EMS systems arriving relatively later at the patients side (RTR < 70%) initiate CPR less frequently and admit fewer patients alive to hospital (calculated per 100,000 inhabitants/year) (CPR incidence (1/100,000 inhabitants/year) RTR > 70% = 57.2 vs RTR < 70% = 36.1, OR = 1.586 (99% CI = 1.383 to 1.819); *P *< 0.01) (admitted to hospital with ROSC (1/100,000 inhabitants/year) RTR > 70% = 24.4 vs RTR < 70% = 15.6, OR = 1.57 (99% CI = 1.274 to 1.935); *P *< 0.01). Using ROSC rate and the multivariate RACA score to predict outcomes, we found that the two groups did not differ, but ROSC rates were higher than predicted in both groups (ROSC RTR > 70% = 46.6% vs RTR < 70% = 47.3%, OR = 0.971 (95% CI = 0.787 to 1.196); P = n.s.) (ROSC RACA RTR > 70% = 42.4% vs RTR < 70% = 39.5%, OR = 1.127 (95% CI = 0.911 to 1.395); *P *= n.s.)

**Conclusion:**

This study demonstrates that, on the level of EMS systems, faster ones more often initiate CPR and increase the number of patients admitted to hospital alive. Furthermore, we show that, with very different approaches, all centres that adhere to and are intensely trained according to the 2005 European Resuscitation Council guidelines are superior and, on the basis of international comparisons, achieve excellent success rates following CPR.

## Introduction

Sudden cardiac arrest is one of the most frequent causes of death in the world. In the United States and Europe, about 300,000 and 450,000 people, respectively, meet this fate [1,2]. Males are affected markedly more frequently than females: The ratio is 4.1:2.7 [3]. In Germany, data derived from the World Health Organisation's MONICA registry (Multinational Monitoring of Trends and Determinants of Cardiovascular Disease) show a predicted incidence 123/100,000 inhabitants/year in the 35- to 64-year-old age group [4,5], whereas in the European Union (EU), per 100,000 inhabitants, resuscitation attempts are performed to treat only about 55 patients [1,6-9]. Thus, in the EU, with about 500 million inhabitants, more than 275,000 resuscitation attempts are made annually. However, more than half of the patients in sudden cardiac arrest die without any resuscitation attempt because the event occurs unwitnessed or the emergency medical service (EMS) team, owing to statutory requirements, arrives too late at the patient's site and only can declare the patient's death. Out-of-hospital cardiopulmonary resuscitation (CPR) poses a huge challenge to emergency medical services because sudden cardiac death is a particularly time-critical event. Additionally, successful management requires a complex and target-oriented response of all acting persons and the entire chain of survival, from dispatch centre personnel to the hospital team.

To improve treatment, the International Liaison Committee on Resuscitation (ILCOR), or rather the European Resuscitation Council (ERC), publishes new resuscitation guidelines regularly every 5 years, with the latest one being released in October 2010 [10-17]. To develop these guidelines, experts in various scientific fields screen and evaluate current studies, amongst which are studies concerning 'telephone-guided CPR' [18-20], therapeutic hypothermia [21-24] or vasopressin treatment [25-27]. Following publication of the guidelines, it is essential to teach and subsequently implement them into EMS systems.

In highly qualified EMS systems, including well-trained emergency physicians, spontaneous circulation may be restored in up to 53% of patients at least until admission to hospital [1,9,28]. Discharge rates in these EMS systems are reported to be 14% to 20%, and 1-year survival rates can reach up to 12%. The 10-year survival rates of patients discharged from hospital may reach 46% [1,9,28,29]. Compared with these highly qualified EMS systems, markedly lower success rates are observed in other EMS systems, with only 9% to 12% of patients being admitted to hospital and only 1% to 3% being discharged from hospital with good neurological outcomes [1,8,9,28-30].

These data clearly show that there are considerable differences between EMS systems concerning treatment success following cardiac arrest and resuscitation, although in all systems the current international guidelines for resuscitation are used [1,6,8,9,28]. It is therefore essential to analyse the reasons for these differences. However, few studies have been published correlating resuscitation results with known influencing factors such as response times, qualifications of team members, actions taken during resuscitation and quality management procedures.

Not least for this purpose, the German Society for Anaesthesiology and Intensive Care Medicine (Deutsche Gesellschaft für Anästhesiologie und Intensivmedizin (DGAI)) has set up the German Resuscitation Registry (GRR), which was officially implemented in 2007 [31,32]. In this study, we investigated the impact of response time reliability (RTR) on CPR incidence and resuscitation success using return of spontaneous circulation (ROSC) after cardiac arrest (RACA) score and data from seven German EMS systems participating in the GRR.

## Materials and methods

### Participating centres

The following are the participating EMS systems studied: (1) the city of Bonn, (2) the county hospital 'Klinik am Eichert', Göppingen, (3) the county of Gütersloh, (4) the city of Münster, (5) the county of Tübingen, (6) the county of Rendsburg-Eckernförde and (7) the region of Marburg. In all of these seven EMS systems, well-trained emergency physicians are responsible for the resuscitation procedures performed at the emergency site. The EMS systems named and the scientific advisory board of the resuscitation registry of the DGAI approved the participation in and the performance of this comparison (trial no. 02/2011 ReaReg).

### The German Resuscitation Registry

The nationwide interdisciplinary GRR run by the DGAI, based on national and international recommendations (MIND2, Utstein-style guidelines and reporting templates, European Registry of Cardiac Arrest (EuReCa) and ILCOR Guidelines), is described in detail elsewhere [2,31,32]. It centrally collects data from 84 participating centres. We analysed the quality reports of the seven centres that participated in our study.

### Inclusion criteria for the resuscitation registry

Patients in whom out-of-hospital cardiac arrest (OHCA) had been diagnosed and a resuscitation attempt had been performed were included independently of the reason for OHCA. Great value was set upon all EMS treatment details and all corresponding data being completely transferred into the resuscitation registry, making it possible to calculate the resuscitation incidence.

### Study period

The study period comprises the years 2006 to 2009. However, the single EMS systems reported periods of various lengths. The centres provided complete data sets for at least one entire calendar year.

### Structural process and quality of results

According to requirements of the resuscitation registry, the following structural quality data of the EMS systems were recorded: the population served; the service area; population density; unit-hours of advanced cardiac life support (ALS) or basic life support (BLS), unit hours/100,000 inhabitants/year and unit-hours per service area. A 'unit-hour' is defined as a fully equipped response unit's being on a response or waiting for a response for 1 hour.

The following data regarding process quality were recorded: RTR (rate (%) of first vehicles' arriving at the scene within 8 minutes of the call to the dispatch centre); response time interval defined as the time from the call's being received in the dispatch centre until the arrival of the first ambulance at the scene, calculated using the time stamps available with dispatch technology; rate (%) of EMS CPR started within 8 minutes of the call to the dispatch centre; rate of dispatch under triage (no ALS unit (emergency physician-staffed) for the first alert); rate of special CPR measures (active compression decompression (ACD) CPR, load-distributing band (LDB) CPR, and CPR feedback); medical director and quality assurance programme; and rate of prehospital cooling to achieve therapeutic prehospital hypothermia.

According to the Utstein-style recommendations and requirements of the resuscitation registry, the following data regarding patients and the circumstances of their cardiac arrest were collected: cause of cardiac arrest, age, gender, witnessed by a bystander or EMS personnel, CPR performed by a bystander, location of cardiac arrest and first electrocardiogram (ECG) rhythm.

According to the Utstein-style recommendations and requirements of the resuscitation registry, the following data regarding resuscitation outcomes were recorded: ROSC, admitted to hospital with spontaneous circulation, 24-hour survival rate and hospital discharge rate.

Resuscitation procedures were performed according to the 2005 ILCOR guidelines. If not already initiated by bystanders or first responders, the resuscitation attempt was started or continued by the first team that arrived at the site (BLS or ALS unit).

### Incidence of CPR attempts and resuscitation success

For the calculation of incidence, the number of patients in whom CPR was attempted and/or ROSC status and/or were admitted to hospital were counted. The incidence data refer to cases per 100,000 inhabitants of the respective EMS system areas per year.

### Quality of CPR performance

To compare the quality of CPR performance between EMS systems, we used the percentage survival rates of all patients andespecially the subgroup of patients found in ventricular fibrillation (VF) and/or ventricular tachycardia (VT) (cardiac origin), according to the Utstein-style recommendations. To compare EMS systems, we used hospital admission rates and not discharge rates, because postresuscitation care has a significant impact on discharge rate.

The second method we used to compare the quality of CPR performance within the centres was the calculation of the RACA scores [33], which predict the ROSC rate (%), including the following factors: age, gender, cause of cardiac arrest, location of cardiac arrest, first ECG rhythm, CPR performed by a bystander and time of EMS arrival.

### Statistics and analysis

Data were processed using Excel XP software (Microsoft Corp, Redmond, WA, USA). Distributions are reported as absolute numbers and percentages. Statistical analyses were performed using χ^2 ^and *t*-tests, respectively, with a difference of *P *< 0.05 considered statistically significant. The Bonferroni correction was used to neutralise the α error in connection with multiple paired comparisons. For analysis of the influence of the RTR on CPR incidence and success, we grouped the EMS systems by RTR using a cut-off level of 70%. Odds ratios and confidence intervals were calculated to compare the two groups of EMS systems. The analysis of numeric variables is specified with means and standard deviations using the SPSS version 14.0 statistical software package (SPSS Inc, Chicago, IL, USA).

### Ethics committee vote

The design and publication of this study were approved by the scientific committee of the GRR in compliance with current publication guidelines. Patients' informed consent was waived by the ethics committee of the University of Cologne Faculty of Medicine (Kerpener Strasse 62, D-50937 Cologne, Germany), due to the fact thatanalysis of anonymous data collection for quality management was not considered necessary to be approved.

## Results

### Sociodemographic characteristics

The EMS systems of Bonn and Münster serve big-city population structures with a high population density, whereas the EMS systems in Rendsburg-Eckernförde, Marburg and Tübingen cover rural areas with a low population density (Table 1). Göppingen and Gütersloh have both urban and rural areas within their EMS regions. Time periods ranging from 12 months (Marburg) and 44 months (Göppingen) were analysed. The entire study period was from 1 May 2006 to 31 December 2009, and during that time 2,330 resuscitation attempts were started.

**Table 1:** Sociodemographic characteristics of the centres

Sociodemographic characteristics	Bonn	Göppingen	Gütersloh	Marburg	Münster	Rendsburg-Eckernförde	Tübingen	*P*-value	Totals or averages
Served population, (*n*)	315,000	192,000	319,732	251,800	280,199	272,488	218,692		1,849,911
Service area (km^2^)	141.0	354.0	864.0	1,262.6	302.9	2,185.9	519.2		5,629.6
Population density (1/km^2^)	2,234.0	542.4	370.1	199.4	925.0	124.7	421.2		328.6
Time frame	1 Jan 2007 to 31 Dec 2009	1 May 2006 to 31 Dec 2009	1 Nov 2007 to 31 Dec 2009	1 Jan 2008 to 31 Dec 2008	1 Jun 2007 to 31 Dec 2009	1 Jan 2006 31 Dec 2007	1 Jan 2007 to 31 Dec 2009		
Person-years	945,000	704,000	692,753	251,800	723,847	544,976	656,076		4,518,452
CPR attempted (*n*)	533	399	410	164	391	196	237		2,330
CPR incidence (1/year/100,000 inhabitants)	56.4	56.7	59.2	65.1	54.0	36.0	36.1	< 0.001	50.6
Rate of first vehicle's reaching emergency patient within 8 minutes (%)	95.5	70.4	77.9	79.8	90.0	65.6	62.0	< 0.001	80.0
Rate of CPR started within 8 minutes (%)	67.9	60.6	57.6	57.9	64.2	56.0	53.0	< 0.001	60.3
Witnessed (%)	64.2	58.1	58.3	67.7	59.6	65.8	49.4	< 0.001	60.2
Witnessed by bystander (%)	53.8	45.6	47.1	58.5	53.2	59.7	38.4	< 0.001	50.4
CPR performed by bystander (%)	23.3	10.0	20.2	17.1	28.6	24.0	1.3	< 0.001	18.8
Witnessed and CPR performed by EMS (%)	10.3	12.5	11.2	9.1	6.4	6.1	11.0	0.09	9.8
Males (%)	64.4	66.9	66.1	68.3	68.0	71.9	66.7	0.64	66.9
Mean age (years)	66.9	68.9	67.9	65.9	67.4	65.2	65.3		67.1
Median age (years)	70.6	73.0	70.9	69.4	70.2	68.6	70.0		70.2
Age (SD)	17.7	16.1	16.6	16.6	17.0	16.5	19.8		17.2
> 65 years old (%)	65.5	71.4	67.3	57.9	63.4	62.2	66.2	< 0.05	
Location of cardiac arrest (%)									
Home	70.5	68.2	77.6	71.3	68.0	69.4	69.2	0.05	70.8
Public	17.4	17.3	16.6	15.9	22.0	20.9	18.1	0.37	18.3
Other	12.0	14.5	5.9	12.8	10.0	9.7	12.7	< 0.01	10.9

CPR = cardiopulmonary resuscitation; EMS = emergency medical service. Data are for service areas and populations served by the EMS systems. Unit hours = fully equipped response unit on a response or waiting for a response for 1 hour. *P*-values were calculated by *t*-test or χ^2 ^test, with significance set at *P *< 0.05.

### Response time reliability

**Figure 1:**
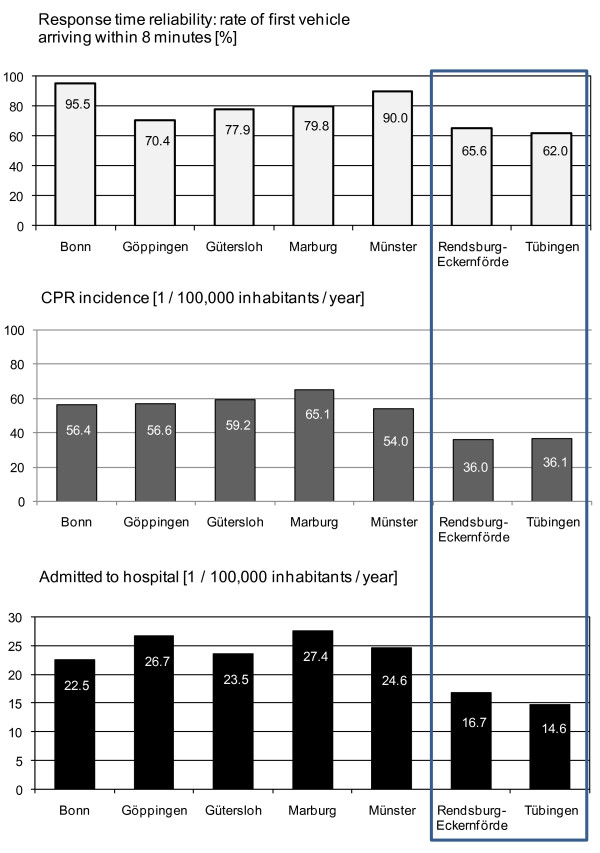
**Response time reliability rate of first vehicle stop within 8 minutes of dispatch, cardiopulmonary resuscitation (CPR) incidence (1/100,000 inhabitants/year) and patients admitted to the hospital (1/100,000 inhabitants/year)**.

In Tübingen and Rendsburg-Eckernförde, only 62.0% and 65.6%, respectively, of patients were reached by the EMS within 8 minutes after being alerted (Table 1 and Figure 1). In the other centres, 70.4% to 95.5% of the patients were treated by the EMS within this period of time. In the big-city areas of Bonn and Münster, about 90% of the patients were reached by the first ambulance within 8 minutes after the EMS was alerted. This performance is much faster than that in the other five EMS systems (*P *< 0.001). Accordingly, in Bonn and Münster, resuscitation attempts were started the earliest (67.9% and 64.2%, respectively, within 8 minutes after being alerted; *P *< 0.001).

### CPR incidence

The calculated incidence of sudden cardiac death followed by resuscitation attempt was between 36.0 and 65.1/100,000 inhabitants/year (Table 1 and Figure 1). In two regions (Rendsburg-Eckernförde and Tübingen), the CPR incidence amounted to 36.0 and 36.1/100,000 inhabitants/year, respectively. In the other regions with shorter response intervals, a minimum of 54 and up to 65.1 resuscitation attempts/100,000 inhabitants/year were registered (*P *< 0.001).

### Circumstances of cardiac arrest

Cardiac arrest was witnessed in about 60% of patients, most rarely in Tübingen (49.4%) and most often in Marburg (67.7%) (*P *< 0.001) (Table 1). In most cases, the witnesses were laypeople or bystanders (38.4% in Tübingen and up to 59.7% in Rendsburg-Eckernförde; *P *< 0.001). EMS personnel were present at the scene when the cardiac arrest occurred less often (6.1% in Rendsburg-Eckernförde and up to 12.5% in Göppingen; *P *= 0.09). In contrast, the rate of bystander CPR attempts was low. Only in a few cases did laypeople start CPR before EMS arrival, even when they had witnessed the person's collapse. The rate of bystander CPR attempts was 1.3% in Tübingen and 28.6% in Münster (*P *< 0.001).

Men more frequently have cardiac arrests than women. A mean of 66.9% of the patients were male, and there were only minor differences between the centres (64.4% to 71.9%; *P *= 0.64). The mean age of patients collected from the different centres was comparable (67.1 ± 17.2 years), with patients being slightly younger in Rendsburg-Eckernförde (65.2 ± 16.5 years) and slightly older in Göppingen (68.9 ± 16.1 years). There were small differences between the centres regarding patients older than 65 years of age (*P *< 0.05). Regarding the site of cardiac arrest, there were small differences between centres. Most collapses occurred in domestic environments (68.0% to 77.6%; *P *= 0.05), in public places (15.9% to 22.0%; *P *= 0.37) and at other sites (5.9% to 14.5%; *P *< 0.01).

### EMS systems, medical treatments and special measures

In all participating centres, the two-tiered system of BLS and ALS units has been established (emergency physician-staffed), meeting at the site of the emergency (Table 2). The availability of EMS teams results from the time during which units are held available. The highest amount of unit-hours/100,000 inhabitants/year was reported by Marburg (54,314 unit-hours) and the lowest was reported by Münster (22,603.2 unit-hours). The lowest amount of unit-hours/service area/year was reported by Rendsburg-Eckernförde (48.1 unit-hours) and the highest was reported by Bonn (723.1 unit-hours).

**Table 2:** EMS systems data

Description of the EMS systems		Bonn	Göppingen	Gütersloh	Marburg	Münster	Rendsburg-Eckernförde	Tübingen	*P*-value	Average
Providers		City of Bonn/fire department	EMS, district of Göppingen, Klinik am Eichert Göppingen	EMS district of Gütersloh	EMS, district of Marburg	City of Münster/fire department	EMS, district of Rendsburg-Eckernförde	EMS, DRK and ASB Tübingen		All
Vehicles	Two-tiered system	Yes	Yes	Yes	Yes	Yes	Yes	Yes		
ALS unit (emergency physician)	Unit-hours (1/year/100,000 inhabitants)	5,561.9	6,463.5	10,959.2	10,436.9	6,252.7	6,429.6	8,011.3	< 0.001	7,773.9
BLS unit	Unit-hours (1/year/100,000 inhabitants)	26,807.6	18,250.0	25,923.0	43,876.9	16,350.5	32,148.2	24,033.8	< 0.001	26,964.8
ALS + BLS unit	Unit-hours (1/year/100,000 inhabitants)	32,369.5	24,713.5	36,882.1	54,313.7	22,603.2	38,577.8	32,045.1	< 0.001	34,738.6
ALS + BLS unit	Unit-hours/year/area (hours/km^2^)	723.1	134.0	136.5	108.3	209.1	48.1	135.0	< 0.001	114.2
Quality assurance	Training programme	RA + RS: 30 hours/year	RA + RS: 30 hours/year NA: 12 hours/year	RA + RS: 30 hours/year NA: 8 hours/year	RA + RS: 38 hours/year NA: 8 hours/year	RA + RS: 30 hours/year NA: 4 hours/year	RA + RS: 30 hours/year	RA + RS: 30 hours/year NA: 12 hours/year		
	Additional emergency physician requested by ambulance crew	9.0	11.5	8.8	11.6	17.9	8.7	3.8	< 0.001	10.5
Equipment	LDB CPR (%)	15.4	0.0	0.0	0.0	0.3	0.0	0.0	< 0.001	3.6
	ACD CPR (%)	4.5	42.6	0.0	5.5	7.2	0.0	6.8	< 0.001	10.6
	Feedback system (%)	0.0	0.0	0.0	1.8	90.3	0.0	0.0	< 0.001	15.3
Prehospital cooling	Cooling of ROSC patients (%)	72.0	50.3	40.2	33.3	64.0	1.0	7.9	< 0.001	46.2

ACD = active compression decompression; ALS unit = NEF (Notarzteinsatzfahrzeug): emergency vehicle providing advanced life support, including an emergency physician; ASB = Arbeiter Samariter Bund; BLS unit = RTW (Rettungswagen): emergency vehicle providing basic life support and defibrillation, without an emergency physician; CPR = cardiopulmonary resuscitation; DRK = Deutsches Rotes Kruez (German Red Cross); EMS = emergency medical service; LDB = load-distributing band; NA: Notarzt = emergency physician; RA: Rettungsassistent = PM: paramedic; ROSC = return of spontaneous circulation; RS: Rettungssanitäter = EMT: emergency medical technician. *P*-values were calculated by *t*-test or χ^2 ^test, with significance set at *P *< 0.05.

It is essential that the staff of dispatch centres identify cardiac arrest victims correctly so that BLS and ALS units are sent out immediately. If an ALS unit has to be requested later by the BLS unit after the BLS unit's arrival at the scene, a deficit in identifying cardiac arrest results (under triage by the dispatch centre). The rate of under triage was different between Münster (17.9%) and Tübingen (3.8%) (*P *< 0.001).

In some centres, additional CPR devices are used besides the normal equipment. In Bonn, for example, in 15.4% of all cases, mechanical resuscitation was performed with a LDB CPR device. In Münster, a CPR feedback system was used for 90.3% of the patients. ACD CPR was not available in Gütersloh and Rendsburg-Eckernförde, whereas the other centres, most frequently in Göppingen (42.6%), used this system.

All centres have implemented regular CPR training, but with differences concerning intervals and intensity. For emergency physicians, the training is done partly on a voluntary basis. The recommended induction of mild hypothermia following resuscitation and ROSC was performed most frequently in Bonn (72.0%) and Münster (64.0%) and markedly less often in Tübingen (7.9%) and Rendsburg-Eckernförde (only 1.0%) (*P *< 0.001).

### CPR success and clinical outcomes

Table 3 shows the survival rates following sudden cardiac arrest and resuscitation at the seven EMS systems (see also Figure 1). The survival rates were calculated by two different methods: (1) The survival rates were calculated as percentages for all patients and the respective Utstein subgroups, and (2) the absolute number of survivors/100,000 inhabitants/year are reported. The frequency of ROSC and hospital admissions with ROSC could be determined for all centres. The entire 24-hour survival data could be determined for Bonn, Göppingen, Gütersloh, Marburg, Münster and Tübingen, but not for Rendsburg-Eckernförde. Discharge rates were completely recorded only for Göppingen, Gütersloh and Marburg. Overall, 2,330 patients were resuscitated in the seven EMS systems. In 46.7%, spontaneous circulation could be restored, 42.8% of the patients were admitted to a hospital with ROSC, 30.7% survived for 24 hours, and 15.4% were discharged alive.

**Table 3:** Clinical outcomes

Clinical outcome		Bonn		Göppingen			Gütersloh			Marburg			Münster			Rendsburg-Eckernförde			Tübingen			p value (1/Y/100.000 I)	p value (%)
			
	[n]	[1/Y/100, 000 I	%	[n]	[1/Y/100, 000 I	%	[n]	[1/Y/100, 000 I	%	[n]	[1/Y/100, 000 I	%	[n]	[1/Y/100, 000 I	%	[n]	[1/Y/100, 000 I	%	[n]	[1/Y/100, 000 I	%		
**All (cardiac and non-cardiac)**	533	56,4	100,0	399	56,6	100,0	410	59,2	100,0	164	65,1	100,0	391	54,0	100,0	196	36,0	100,0	237	36,1	100,0	**< 0.001**	
any ROSC	250	26,5	46,9	191	27,1	47,9	179	25,8	43,7	75	29,8	45,7	189	26,1	48,3	104	19,1	53,1	101	15,4	42,6	**< 0.001**	**0,32**
any ROSC CI 95%			41,9/51,5			43,5/55,2			38,7/52,1			41,4/61,4			44,0/56,4			45,7/63,8			37,4/54,3		
RACA Score			41,8			39,7			42,4			45,5			44,7			42,4			37,1		
difference significant			y			y			n			n			n			y			y		
																							
admitted to hospital	213	22,5	40,0	188	26,7	47,1	163	23,5	39,8	69	27,4	42,1	178	24,6	45,5	91	16,7	46,4	96	14,6	40,5	**< 0.001**	**0,17**
24 hours survival	141	14,9	26,5	121	17,2	30,3	109	15,7	26,6	40	15,9	24,4	59	8,1	15,1	n.d.			56	8,5	23,6	**< 0.001**	**< 0.001**
discharged alive	n.d.			56	8,0	13,8	68	9,8	16,6	27	10,7	16,5	n.d.			n.d.			n.d.			**0,30**	**0,50**
																							
**First rhythm VF/VT (all)**	141	14,9	26,5	105	14,9	26,3	99	14,3	24,1	55	21,8	33,5	119	16,4	30,4	78	14,3	39,8	64	9,8	27,0	**< 0.01**	**< 0.01**
																							
**VF/VT (cardiac)**	125	13,2	23,5	94	13,3	23,6	79	11,4	19,3	44	17,5	26,8	95	13,1	24,3	63	11,6	32,1	57	8,7	24,1	**< 0.05**	**< 0.05**
any ROSC	88	9,3	70,4	68	9,6	72,3	53	7,6	67,1	33	13,1	75,0	70	9,7	73,7	47	8,6	74,6	34	5,2	59,6	**< 0.01**	**0,51**
admitted to hospital	77	8,1	61,6	68	9,6	72,3	47	6,8	59,5	32	12,7	72,7	66	9,1	69,5	43	7,9	68,3	33	5,0	57,9	**< 0.01**	**0,28**
24 hours survival	58	6,1	46,4	52	7,4	55,3	38	5,5	48,1	n.d.			25	3,4	26,3	5	3,4	26,3	23	3,5	40,4	**< 0.001**	**< 0.001**
																							
**Asystoly (cardiac)**	133	14,1	25,0	128	18,2	32,1	94	13,6	22,9	33	13,1	20,1	91	12,6	23,3	52	9,6	26,5	62	9,5	26,2	**< 0.001**	**< 0.05**
any ROSC	43	4,6	32,3	42	6,0	32,8	24	3,5	25,5	9	3,6	27,3	26	3,6	28,6	16	2,9	30,8	15	2,3	24,2	**< 0.05**	**0,82**
admitted to hospital	31	3,3	23,3	42	6,0	32,8	22	3,2	23,4	8	3,2	24,2	23	3,2	25,3	11	2,0	21,2	13	2,0	21,0	**< 0.01**	**0,49**
24 hours survival	18	1,9	13,5	23	3,3	18,0	12	1,7	12,8	n.d.			9	1,2	9,9	1	0,2	1,9	6	0,9	9,7	**< 0.01**	**0,07**
																							
**PEA (cardiac)**	73	7,7	13,7	49	7,0	12,3	30	4,3	7,3	12	4,8	7,3	15	2,1	3,8	3	0,6	1,5	21	3,2	8,9	**< 0.001**	**< 0.001**
any ROSC	32	3,4	43,8	23	3,3	46,9	11	1,6	36,7	5	2,0	41,7	7	1,0	46,7	3	0,6	100,0	11	1,7	52,4	**< 0.001**	**0,92**
admitted to hospital	26	2,8	35,6	22	3,1	44,9	11	1,6	36,7	3	1,2	25,0	6	0,8	40,0	3	0,6	100,0	11	1,7	52,4	**< 0.01**	**0,60**
24 hours survival	18	1,9	24,7	6	0,9	12,2	6	0,9	20,0	n.d.			2	0,3	13,3	n.d.			5	0,8	23,8	**< 0.05**	**0,48**

p-value (1/Y/100,000 I): Comparison of the number of patients in one year per 100,000 Inhabitants of the centre; p-value (%): Comparison of the number of grouped patients regarding to the number of all treated patients;RACA Score: predicted

value of ROSC; VF = ventricular heart flutter; VT = ventricular tachycardia; PEA = pulseless electrical activity; p-value calculated by t-test or chi square test (significant = p < 0.05)

Survival rate differences between the centres were minor. Any ROSC was achieved in 42.6% of patients (Tübingen) and 53.1% of patients (Rendsburg-Eckernförde) (*P *= 0.32). Between 39.8% (Gütersloh) and 47.1% (Göppingen) of patients were admitted to hospital with ROSC (*P *= 0.17). Survival after 24 hours varied from 15.1% (Münster) to 30.3% (Göppingen) (*P *< 0.001). Discharge rates were between 13.8% and 16.6% (*P *= 0.50).

Quality of EMS care should not be measured only by using the 'percentage admission to hospital rate', because a selection bias might influence this rate in both directions. Therefore, in this study, the quality of preclinical care was additionally assessed according to the 'admission rate relative to the population served'.

Regarding CPR incidence, the EMS systems differed significantly. In two of the seven systems, the CPR incidence was below 38/100,000 population/year, and in these two systems, the rate of patients admitted to hospital was significantly lower than in the other centres (*P *< 0.001). In Tübingen and Rendsburg-Eckernförde, only 14.6 and 16.7 patients/100,000 population/year, respectively, were admitted to hospital following cardiac arrest. In the other five systems, between 22.5 (Bonn) and 27.4 (Marburg) patients/100,000 population/year survived the event to hospital admission (*P *< 0.001).

The quality of EMS care may further be assessed on the basis of the real ROSC rate and the predicted ROSC rate (RACA score [33]). The predicted ROSC rate was, on average, 41.9%, with a minimum of 37.1% in Tübingen and a maximum of 45.5% in Marburg. In all seven centres, the ROSC rate was higher than predicted by the RACA score. In four centres (Bonn, Göppingen, Rendsburg-Eckernförde and Tübingen), the ROSC rate was significantly higher than predicted.

An outcome analysis of subgroups according to the initially recorded cardiac rhythm may further specify the comparison of the centres, eliminating an important influencing factor. For example, among the subgroup of patients with a collapse of cardiac origin found in a shockable initial rhythm (23.9% of all patients), the admission rate was 65.7% and thus considerably higher than that of patients with asystole (25.3%) or pulseless electrical activity (40.4%) (incidence = 7.9 vs 3.3 vs 1.8/100,000 inhabitants/year, respectively). Differences between EMS systems can generally also be found in the subgroup analysis. Following collapse of cardiac origin and shockable rhythm, 72.7% were admitted in Marburg, but only 57.9% were admitted in Tübingen (*P *= 0.28). In Göppingen, 55.3% of the patients were alive 24 hours after the event, but only 26.3% were still alive in Münster and Rendsburg-Eckernförde each (*P *< 0.001).

### Impact of response time reliability on CPR incidence and CPR success

**Figure 2:**
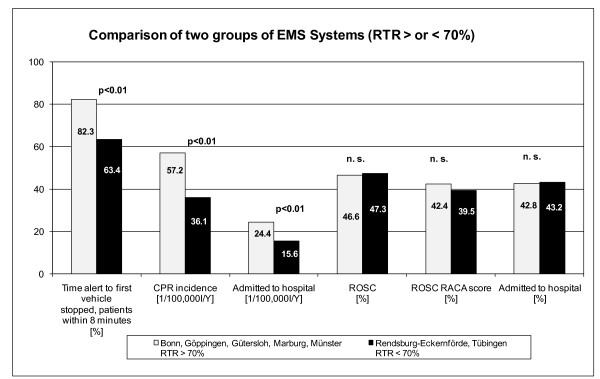
**Comparison of two groups of emergency medical service (EMS) systems grouped by response time reliability (RTR) achieved or not achieved in 70% of dispatches**. RTR calculates the rate of first vehicle arriving within 8 minutes (%). Response time interval was defined from call reception in the dispatch centre until arrival of the first ambulance on scene and was calculated using the time stamps of dispatch technology. Odds ratios, confidence intervals and χ^2 ^tests were used for statistical analysis. n.s. = not significant; CPR = cardiopulmonary resuscitation; RACA = return of spontaneous circulation after cardiac arrest; ROSC = return of spontaneous circulation.

To analyse the impact of RTR on CPR incidence and success, we contrasted the performance of the EMS systems of Bonn, Göppingen, Gütersloh, Marburg and Münster (group 1; RTR > 70%), where > 70% of patients are reached by the first unit within 8 minutes, with the EMS systems of Tübingen and Rendsburg-Eckernförde (group 2; RTR < 70%), where < 70% of the patients are reached within 8 minutes (RTR > 70% = 82.3% vs RTR < 70% = 63.4%, OR = 2.676 (99% CI = 1.93 to 3.711); *P *< 0.01) (Table 4 and Figure 2).

**Table 4:** Comparison of two groups of EMS systems grouped by response time reliability achieved or not achieved in 70% of dispatches

Provider	Bonn, Göppingen, Gütersloh, Marburg and Münster RTR > 70%	Rendsburg-Eckernförde and Tübingen RTR < 70%	*P*-values, ORs and 95% CIs	All seven providers	All other centres in the German Resuscitation Registry
Person-years	3,317,400	1,201,052		4,518,452	1 Jan 2006 to 31 Dec 2009
All patients (cardiac + noncardiac) (*n*)	1,897	433		2,330	4,624
Time from alert until first vehicle stopped (*n*)					
Patients with time	1,860	347			
Patients within 8 minutes	1,530	220			
Statistics	82.3%	63.4%	*P *< 0.01	79.3%	73.6%
			OR = 2.676		
			99% CI = 1.93 to 3.711		
CPR incidence (1/100,000 inhabitants/year)	57.2%	36.1%	*P *< 0.01	51.6%	n.d.
			OR = 1.586		
			99% CI = 1.383 to 1.819		
ROSC (*n*)	884	205			
ROSC (1/100,000 inhabitants/year)	26.6%	17.1%	*P *< 0.01	24.1%	n.d.
			OR = 1.561		
			99% CI = 1.279 to 1.906		
Admitted to hospital (*n*)	811	187			
Admitted to hospital (1/100,000 inhabitants/year)	24.4%	15.6%	*P *< 0.01	22.1%	n.d.
			OR = 1.57		
			99% CI = 1.274 to 1.935		
ROSC RACA (*n*)	804	171			
ROSC RACA	42.4%	39.5%	*P *= n.s.	41.9%	n.d.
			OR = 1.127		
			95% CI = 0.911 to 1.395		
ROSC	46.6%	47.3%	*P *= n.s.	46.7%	37.9%
			OR = 0.971		
			95% CI = 0.787 to 1.196		
Admitted to hospital	42.75%	43.19%	*P *= n.s.	42.83%	32.7%
			OR = 0.982		
			95% CI = 0.878 to 1.116		
RTR > 70%: ROSC vs RACA ROSC	884/1,897 vs 804/1,897		*P *< 0.01		
			OR = 1.186		
			99% CI = 1.002 to 1.404		
RTR < 70%: ROSC vs RACA ROSC		205/433 vs 171/433	*P *< 0.05		
			OR = 1.378		
			95% CI = 1.052 to 1.804		

CPR = cardiopulmonary resuscitation; n.d. = not determined; n.s. = not significant; RACA = return of spontaneous circulation after cardiac arrest; ROSC = return of spontaneous circulation; RTR = response time reliability. RTR is the rate of the first vehicle's arriving at the scene within 8 minutes (%). The response time interval was defined as the time from when a call was received in the dispatch centre until the arrival of the first ambulance at the scene. It was calculated using the time stamps of dispatch technology. Odds ratios, confidence intervals and χ^2 ^tests were used for statistical analysis.

In faster EMS systems with RTR > 70% (group 1), CPR incidence was significantly higher than in group 2 (CPR incidence (1/100,000 inhabitants/year) RTR > 70% = 57.2 vs RTR < 70% = 36.1, OR = 1.586 (99% CI = 1.383 to 1.819); *P *< 0.01) and more patients with ROSC were admitted to hospital (admitted to hospital (1/100,000 inhabitants/year) RTR > 70% = 24.4 vs RTR < 70% = 15.6, OR = 1.57 (99% CI = 1.274 to 1.935); *P *< 0.01). However, these two groups did not differ in 'percentage CPR success rates' (ROSC RTR > 70% = 46.6% vs RTR < 70% = 47.3%, OR = 0.971 (95% CI = 0.787 to 1.196); P = n.s.) (admitted to hospital RTR > 70% = 42.8% vs RTR < 70% = 43.2%, OR = 0.982 (95% CI = 0.878 to 1.116); P = n.s.). On the basis of using the multivariate RACA score to predict outcome, the two groups did not differ, but ROSC rates were higher than predicted in both groups (ROSC RACA RTR > 70% = 42.4% vs RTR < 70% = 39.5%, OR = 1.127 (95% CI = 0.911 to 1.395); *P *= n.s.).

## Discussion

This study demonstrates for the first time a relation between the RTR, CPR incidence and resuscitation success rate for sudden cardiac arrest in Germany (Tables 3 and 4 and Figures 1 and 2). Our study clearly shows that the EMS systems with the longest response intervals have the lowest CPR incidence and CPR success rates, calculated per 1/100,000 inhabitants/year.

It is noteworthy that the 'percentage ROSC rate' and the 'admission to hospital rate', which are usually used to compare EMS systems, did not differ between both groups and thus seem to be weak indicators of the performance of EMS systems (Figure 2). In addition, the RTR seems to be a particularly important influencing factor. On the one hand, it affects the frequency of resuscitation attempts by an EMS system; on the other hand, it affects the resuscitation success related to the population served. In this study, the time interval from when the call is received until the arrival of the first ambulance at the scene was used to calculate, consistently for all centres, the RTR in resuscitation missions. The rate of patients reached within 8 minutes of the call was determined. This time interval corresponds largely to the national standard for response times in the United Kingdom, whereas in Germany, owing to different state laws regarding EMS, there is no nationwide standard. According to the heterogeneous legal requirements, the best RTRs were found in the most densely populated areas (Bonn and Münster), with 90% of the patients reached by the first ambulance within 8 minutes after the call. It is remarkable that in the very rural EMS system of Marburg, which has the second lowest population density, 79.8% of the patients were reached within 8 minutes after the call. This success is explained by high number of EMS vehicles and unit-hours in Marburg. In contrast to the EMS system of Rendsburg-Eckernförde, which also provides service in a rural area, Marburg reached 108.3 unit-hours/km^2 ^service area, which is more than twice the provided unit-hours in Rendsburg-Eckernförde.

A high RTR regularly shortens the interval without treatment, so professional resuscitation attempts may be initiated earlier. In other regions, this leads to improved admission and survival rates as described by Hollenberg *et al. *[34], who compared the resuscitation success rates of Gothenburg and Stockholm (admission rates 30% vs 16%). Vukmir *et al. *[35] showed that more patients survive when it is possible to initiate resuscitation attempts within 8 minutes of the call or not (56 vs 32 patients). Our study supports the demand for a standardised response time interval for the arriving first vehicle and RTR > 70%, meaning that > 70% of the patients should regularly be reached within 8 minutes after the call.

Because regional state laws in Germany differ, response intervals are defined differently and healthcare funds provide financial means to reach only the respective standards. Thus, a German EMS system can realise a response interval standard only within a given legislative and financial framework. To compare the quality of EMS care under these conditions, further indicators must be considered. The survival rates following cardiac arrest are, in addition to other factors, influenced by techniques and quality of BLS [36,37], ALS [38-41] and postresuscitation care [30,42-44]. Therefore, in our study, the quality of EMS care was analysed by additionally assessing 'percentage survival rates', that is, ROSC and admission to hospital, of the total population and subgroups defined beforehand, as well as in comparison to a predictive value (RACA score) [33]. Table 4 shows that both groups of EMS systems could achieve higher ROSC rates than predicted by the RACA score, but did not differ regarding the 'percentage survival rates'. This means that (1) all seven EMS centres studies belong to the the best performing systems in the GRR, and (2) a lower CPR incidence does not lead to a positive selection of 'good risks'. The first statement is additionally supported by a comparison with the admission rates from the GRR, because all seven centres performed better than the other centres participating in the GRR, with, on average, 42.8% vs 32.7% of patients being admitted to hospital.

There might be various reasons for the superior resuscitation results of the seven participating EMS systems that we studied. It is well-known that both a collapse in public and a witnessed collapse improve the chances of surviving an OHCA [9]. However, in this respect, there were no differences between the seven centres participating in our study and the total GRR participants (witnessed = 60.2% vs 61.6%, collapse in public = 18.3% vs 18.2%). The results cannot be explained by the rates of bystander CPR, which were 18.8% in the seven participating centres in our study and 18.5% in the total GRR. It is remarkable that in Germany bystanders too rarely initiate CPR before EMS arrival, even when they witness the collapse. The positive influence of bystander CPR on survival rate has been demonstrated frequently [45-47]. Previous studies have shown similar setups in German and European systems [9,48]. One reason for the low rate of bystander CPR in Germany may be that > 70% of the events occur at home and that it is usually elderly people who are affected and live alone or with an elderly partner who is unable to perform BLS spontaneously. As a consequence, the approach of telephone-guided CPR should urgently be intensified in the EMS systems that we studied and in Germany generally.

The comparatively high survival rates in the seven analysed centres in our study may be explained by the higher rate of patients found in a shockable rhythm (rate of VF/VT = 28.4% vs 23.1% in the entire GRR; *P *< 0.001). Therapeutic hypothermia following ROSC was induced in 46.2% of the patients in the seven centres in our study, but only in 13.7% of all patients in the GRR (*P *< 0.001).

Special efforts are made in all seven centres studied regarding CPR training in general, particularly BLS CPR. This is reflected by the fact that, in three centres, intensive training is provided in the use of special supportive devices, which are used extensively. Bonn has established LDB CPR use [37,49], ACD-CPR is applied in connection with an impedance valve in Göppingen [50] and, after intensive training and continuous scientific evaluation, a CPR feedback system is regularly used in Münster [51,52]. In this study, we found no evidence that using these mechanical or feedback devices increases CPR success. However, as the data derived from the remaining participating centres show, excellent results are possible by applying only committed manual CPR.

### Limitations

The relationship between RTR, CPR incidence and hospital admission rate in this study including seven EMS systems is obvious, but needs to be examined in more detail on the basis of a greater number of included EMS systems.

## Conclusions

The results of this study demonstrate that, with regard to the level of EMS systems, the faster ones more often initiate CPR and increase the number of patients admitted to hospital alive. Furthermore, we have shown that with the use of very different approaches, all EMS systems that adhere to and provide intensive training based on the 2005 ERC guidelines, superior and, on the basis of international comparisons, excellent success rates following resuscitation can be achieved. The data derived from the three EMS systems in our study (Göppingen, Gütersloh and Marburg) in which the discharge rates based on 1/100,000 inhabitants/year could be calculated, with results between 8.0 and 10.7/100,000 inhabitants/year, take a top position in Europe (Table 3). Despite these internationally compared excellent results, some potential improvements could be identified for the centres. (1) Change of location of ambulance and emergency physician's stations, implementation of global positioning systems (GPSs) and computer-aided dispatch systems should be used to improve the rate of calls reached within the standardised response time interval. (2) The time interval between EMS arrival and onset of CPR should be shortened. (3) Intensive, required training in BLS should be implemented, especially when mechanical devices are used. (4) Special CPR training for elderly citizens should be required. (5) Awareness should be raised among, and training should be provided to, the general population regarding the importance of bystander CPR. (6) A structured interview of emergency calls and telephone-guided CPR instructions by the dispatch centre should be implemented. (7) Consistent use of a standard operating procedure concerning treatment of hypothermia, starting in the preclinical phase, should be implemented.

## Key messages

• Later arrival of the first EMS unit at the scene decreases the incidence of CPR, the number of patients who reach ROSC and who can be admitted to hospital with ROSC. Therefore, the RTR, that is, the rate of the first vehicle's arriving within 8 minutes after the call is received at the dispatch centre, should be > 70%.

• Changes in the location of ambulance and emergency physician stations, as well as the use of GPS devices and computer-aided dispatching systems, should be implemented to improve the rate of OHCA victims reached within the standardised response time interval.

• Telephone-guided CPR should be introduced to increase the rate of bystander CPR.

• BLS training should be required for use among the general public and special groups of elderly people to reduce no-flow time until EMS arrives to take over CPR.

• For comparison and benchmarking of EMS systems, not only 'percentage survival rates' but also the number of patients with CPR attempted and/or ROSC and/or admitted to hospital, referring to 1/100,000 inhabitants/year of the respective EMS systems, should be calculated.

## Abbreviations

ACD: active compression decompression; ALS: advanced cardiac life support; BLS: basic life support; CPR: cardiopulmonary resuscitation; DGAI: German Society for Anaesthesiology and Intensive Care Medicine (Deutsche Gesellschaft für Anästhesiologie und Intensivmedizin); ECG: electrocardiogram; EMS: emergency medical service; ERC: European Resuscitation Council; EU: European Union; EuReCa: European Registry of Cardiac Arrest; GRR: German Resuscitation Registry; ILCOR: International Liaison Committee on Resuscitation; LDB: load-distributing band; NA: Notarzt (emergency physician); NEF: Notarzteinsatzfahrzeug (emergency vehicle staffed with emergency physicians); OHCA: out-of-hospital cardiac arrest; RA: Rettungsassistent (paramedic); RACA: return of spontaneous circulation after cardiac arrest; ROSC: return of spontaneous circulation; RS: Rettungssanitäter (emergency medical technician); RTR: response time reliability; RTW: Rettungswagen (emergency vehicle staffed with paramedics but no doctors); VF: ventricular heart flutter; VT: ventricular tachycardia.

## Competing interests

JTG, JW, MM and MF are members of the steering committee of the German Resuscitation Registry. All authors declare that they have no competing interests.

## Authors' contributions

JN and JTG made substantial contributions to the study's conception and design and drafted the manuscript. MF conceived the study, participated in its design and coordination and helped to draft the manuscript. JCS, MB, UH, JW, AB, GH, BS, HF, CK, RL and MM were responsible for data collection and quality control in the participating centres and helped to revise the manuscript. JB, BB, PM, JS and MF were involved in the final critical revision of the manuscript. All authors read and approved the final manuscript.
